# Incidental thoracic, hepatic and peritoneal calcifications: a case of Pentastomiasis

**DOI:** 10.1259/bjrcr.20180058

**Published:** 2018-08-02

**Authors:** Richard Flood, Hedvig Karteszi

**Affiliations:** 1 Department of Radiology, North Bristol NHS Trust, Bristol, UK; 2 Department of Radiology, University Hospitals Bristol, Bristol, UK

## Abstract

Incidental findings are not uncommon in radiology. In this case, although the incidental findings could be described as an Aunt Minnie, the patient underwent multiple investigations due to the rarity of the causative parasite. The current literature concerning Pentastomiasis suggests it may become more common in future. Our hope is that this case report will help future patients who present with the radiological pattern described to be more rapidly diagnosed and reassured.

## Clinical presentation and image findings

**Figure 1.**
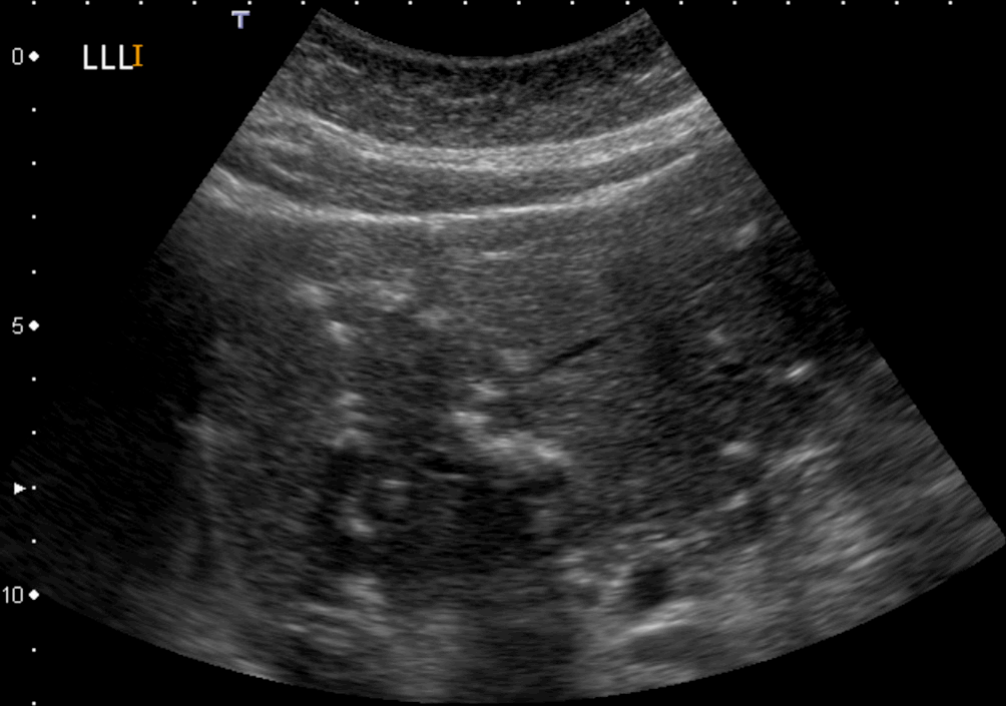
Initial ultrasound of the liver identified multiple presumed calcific, echogenic foci throughout both lobes, predominately in the right lobe.

We report the case of a 49-year-old female of Nigerian origin who was referred for abdominal ultrasound as part of ongoing occupational health testing. The ultrasound was unremarkable except for multiple echogenic foci throughout both lobes of the liver (Figure 1) which were thought to be calcific. Previous imaging and clinical details were not available so a gastroenterology referral was advised.

**Figure 2.**
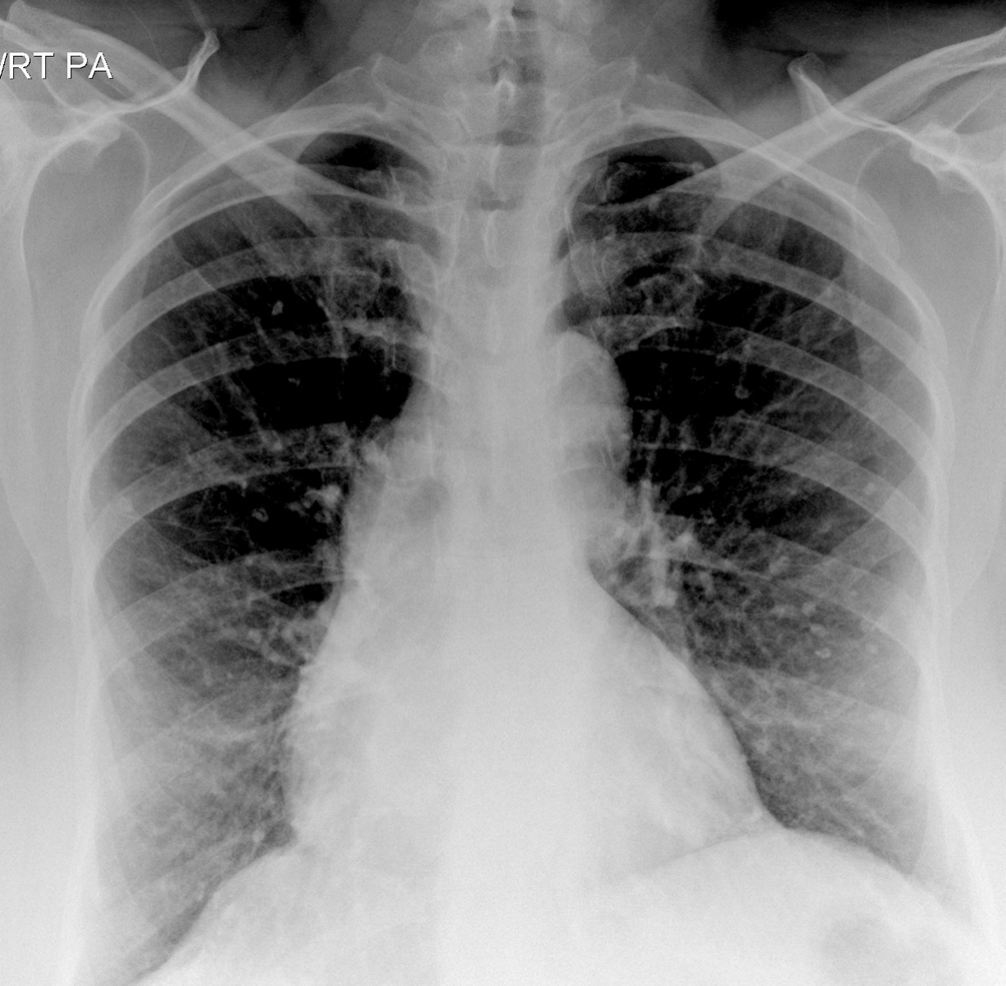
CXR showed multiple c-shaped calcific densities in both lungs. CXR, chest radiograph.

Additional clinical information which was provided with a gastroenterology request for repeat ultrasound and a CXR (chest radiograph) included: known hepatitis B (low viral load), reported right upper quadrant discomfort and “nodules on previous CXR 20 years ago”. Repeat ultrasound appearances were unchanged. The CXR showed multiple calcific densities scattered throughout both lungs (Figure 2). Liver elastography was normal. These studies were followed by a triple phase CT liver scan which showed widespread multifocal calcification in the liver, spleen, mesenteric fat, peritoneal surfaces and lung bases. The right lobe of the liver, where the calcifications were focused, was noted to be atrophic and fibrotic. A differential of “previous inflammatory process *e.g.* TB or parasitic infection” was reported.

**Figure 3.**
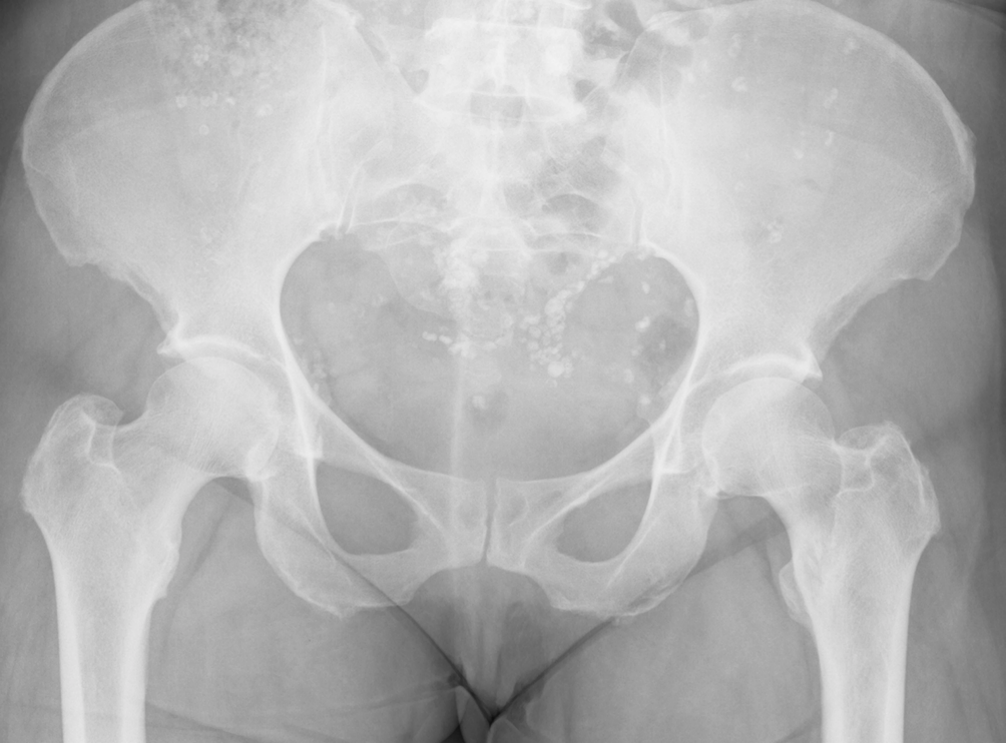
Pelvic radiograph following a fall. Multiple calcific densities throughout the abdominal and peritoneal cavity.

These calcifications were left unexplained and the patient underwent annual ultrasound scans for 5 years. No interval changes were seen over this period. Subsequent history obtained during this follow-up period was of a previous serious childhood illness. A pelvic radiograph was obtained following a fall (Figure 3 *)*. Multiple appendicular radiographs over this period showed no evidence of peripheral calcific lesions.

**Figure 4.**
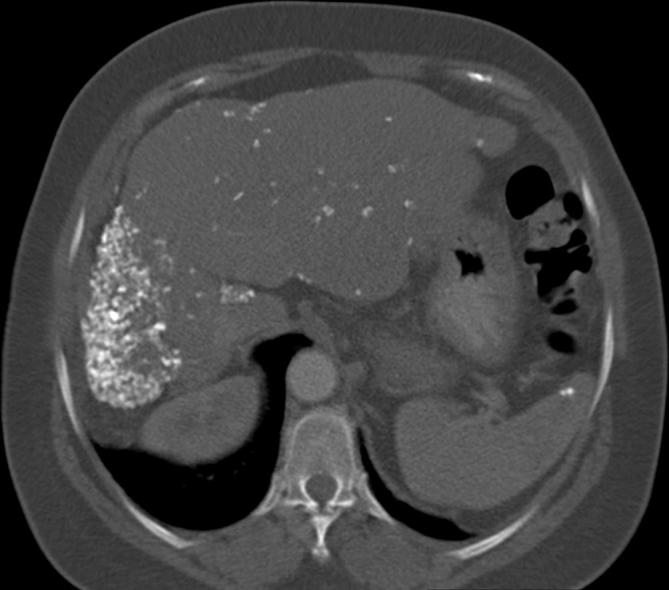
Repeat CT demonstrated unchanged multifocal calcification in the liver and spleen.

**Figure 5.**
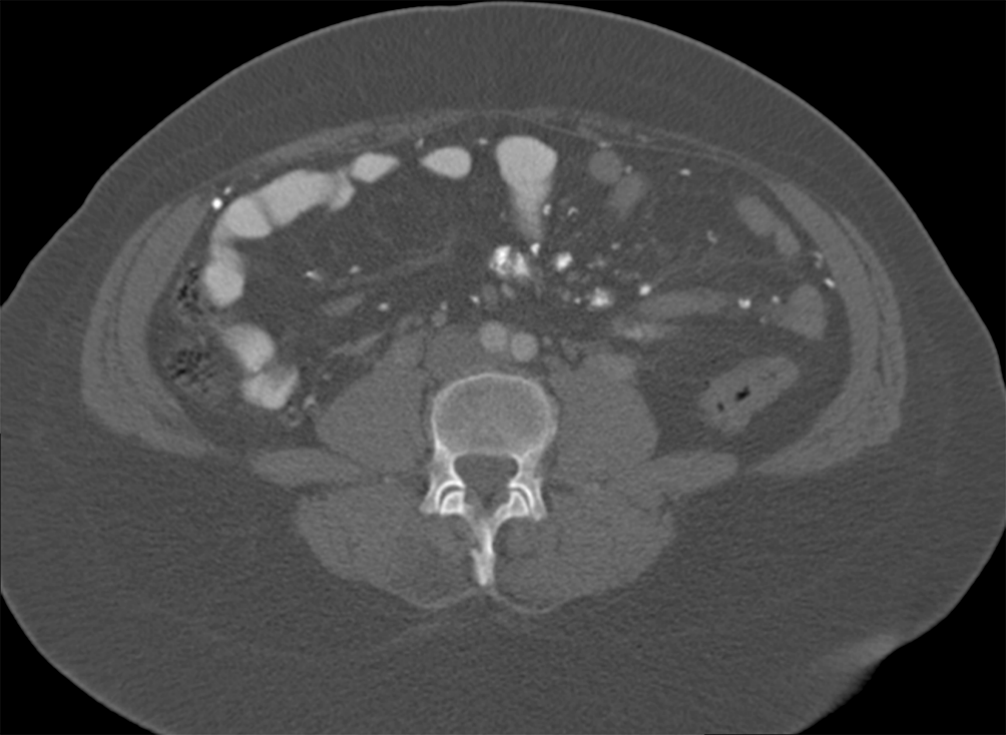
Unchanged calcifications in the peritoneum and mesentery on repeat CT.

In 2017, repeat liver elastography showed a dramatic increase in kPa and concerns about the hepatic calcifications resurfaced so a biopsy was considered. Prior to this a CT thorax, abdomen and pelvis (including triple phase liver) was requested. Again, the appearances were unchanged (Figures 4 and 5). The reporting radiologist of the second CT recognised the pathognomonic radiographic appearances^1–5^ of previous *Armillifer armillatus* infestation. The clinical team were advised elastography would not be accurate due to the extensive hepatic calcifications and that the patient's hepatic fibrosis was due to the previous infestation.^6, 7^

## Discussion

The taxonomy of Pentastomida and the terminology concerning the infections they cause is summarised in Table 1. Whilst there are many species of Pentastomida which can infect humans only two species from the *Armilliferidae* family are radiologically relevant.^4^

**Table 1.** Taxonomy of Pentastomida and the terminology concerning Pentastomida infestation. *Armillifer armillatus* and *Linguatula serrata* account for > 99% of all human Pentastomida infestations

**Subclass**	**Order**	**Family**	**Genus**	**Species**
Pentastomida (pentastomiasis causes pentastomosis)	Porocephalidia (porocephaliasis causes porocephalosis)	Linguatulidae	*Linguatula* (linguatuliasis causes linguatulosis, Halzoun syndrome or Marrara)	*arctica*
*serrata*
Armilliferidae	*Armillifer*	*armillatus*
*moniliformis*
*grandis*

*Armillifer armillatus *and* Armillifer moniliformis* are endoparasites which feed on blood. They have a "screw like" appearance and can reach up to 20 cm in length.^4^

Adult *Armillifer* live in the trachea and bronchi of snakes (definitive hosts) such as pythons and vipers.^4^ Female *Armillifer* release eggs into the snake’s intestinal and respiratory tracts. When intermediate hosts (usually rodents) come in contact with the sputum/faeces of an infected snake or ingest contaminated water, they themselves can become infected. The *Armillifer* eggs hatch in the intestinal tract of their intermediate host and develop into larvae which cross the gut wall, migrate along the peritoneum or pleura and become encysted in various tissues; most commonly liver but also spleen, mesentery, intestine, kidney, omentum, peritoneum and lung.^1,2,4,5,8–10^

The encysted larvae grow slowly and usually die within 2 years. This leads to an inflammatory response which result in absorption or calcification of the dead larvae.^4^

The life cycle is completed if the intermediate host is ingested by a snake whilst it hosts live larvae. When the live larvae reach the snake’s intestinal tract they migrate to the respiratory tract, develop into adult *Armillifer* and the cycle repeats.

Humans (dead end hosts) are infected by coming into contact with sputum/faeces of an infected snake, contaminated water or under cooked snake meat.^4, 5,9,11,12^ It later came to light that the patient described in this case has a close family member who works with bushmeat.

*Armillifer armillatus* is most commonly seen in West Africa, particularly Nigeria.^1–5,8–13^ *Armillifer* moniliformis is more common in Asia.^4^

Most cases are asymptomatic.^10^ In cases which are symptomatic presentation varies according to the tissues affected and can range from fever and abdominal pain^1, 10,11,13^ to intestinal obstruction,^3^ bacterial septicaemia, severe enterocolitis and death.^8^ One study showed *Armillifer armillatus* infection to be the third most common cause of hepatic fibrosis in Nigeria, which was seen in our case and previous cases.^5, 12^

Diagnosis is usually incidental following radiological investigation, autopsy^8, 11^ or laparotomy.^3, 4,10^ Isolation of live larvae allows histological diagnosis. Serological and PCR tests specific to *Armillifer* are possible but not usually available.^4, 13,14^ Patients may have a mild eosinophilia.^2, 4,11^

The radiographic appearances are pathognomonic; multiple crescentic, horseshoe, coiled or comma-shaped calcifications distributed mainly within the upper abdomen and thorax.^1–5^ The size of these calcifications varies from 4 to 8 mm.^4^ Unlike cysticercosis (*Taenia solium* infestation), the musculature is spared in *Armillifer* infestation.^4^ Ultrasound will show multiple hyperechoic lesions in the affected tissues.^2, 5,9,11,13^ It is worth noting not all cases will present in this way, a case of a Pentastomiasis granuloma with the radiographic appearances of hepatic malignancy has previously been reported.^15^ In this case the diagnosis was only reached after surgical resection had been carried out.^15^

Treatment is not required for asymptomatic patients. There is no standard treatment for symptomatic patients but surgery might be indicated in selected cases.^4, 10,13^Praziquantel, albendazole or mebendazole have reportedly been successful at eradicating Pentastomida species.^2, 4,11,13,14,16^

Snake meat is becoming more common at bushmeat markets and consumption is increasing.^14, 16^ As migration increases, radiographic evidence of previous *Armillifer armillatus infestation* may become a more common finding in Western countries.

Incidental discovery of the pathognomonic appearances described has in many cases (including ours) led to multiple further investigations before a diagnosis is reached. We hope this case report will help such patients to be more rapidly diagnosed and reassured.

## Learning points

1. *Armillifer armillatus* is an endoparasite which lives in the respiratory tract of snakes, it is most commonly found in West Africa.
2. Humans can become infected, especially those from West Africa who handle or consume snake meat.
3. It is rarely symptomatic.
4. It has a pathognomonic radiological appearance: multiple crescentic, horseshoe, coiled or comma-shaped calcifications distributed mainly within the upper abdomen and thorax.
5. Treatment is not required for asymptomatic individuals, there is no standard treatment for symptomatic patients.
6. The hunting, selling and consumption of bushmeat is increasing. As migration from Africa increases, the radiographic appearances described may become a more common finding in Western countries.
